# Nanomaterials for Plant Disease Diagnosis and Treatment: A Review

**DOI:** 10.3390/plants13182634

**Published:** 2024-09-20

**Authors:** Luis M. Carrillo-Lopez, Clemente Villanueva-Verduzco, Evert Villanueva-Sánchez, Marja L. Fajardo-Franco, Martín Aguilar-Tlatelpa, Rosa I. Ventura-Aguilar, Ramón Marcos Soto-Hernández

**Affiliations:** 1 Consejo Nacional de Humanidades, Ciencias y Tecnologías-Botánica, Colegio de Postgraduados Campus Montecillo, Carretera Mexico-Texcoco Km. 36.5, Texcoco 56230, Mexico; 2 Departamento de Fitotecnia, Universidad Autónoma Chapingo, Carretera México-Texcoco Km. 38.5, Chapingo 56230, Estado de México, Mexico; clemente@correo.chapingo.mx; 3 Consejo Nacional de Humanidades, Ciencias y Tecnologías-Laboratorio Nacional de Investigación y Servicio Agroalimentario y Forestal, Universidad Autónoma Chapingo, Carretera México-Texcoco Km. 38.5, Chapingo 56230, Estado de México, Mexico; evillanueva@conahcyt.mx; 4 Posgrado en Manejo Sustentable de Recursos Naturales, Universidad Intercultural del Estado de Puebla, Calle Principal a Lipuntlahuaca, Huehuetla 73475, Puebla, Mexico; marjaliza.fajardo@uiep.edu.mx (M.L.F.-F.); martin.aguilar@uiep.edu.mx (M.A.-T.); 5 CONAHCYT-Recursos Genéticos y Productividad-Fruticultura, Colegio de Postgraduados, Campus Montecillo, Carretera Mexico-Texcoco Km. 36.5, Texcoco 56230, Mexico; riventuraag@conahcyt.mx; 6 Botánica, Colegio de Postgraduados, Campus Montecillo, Carretera Mexico-Texcoco Km. 36.5, Texcoco 56230, Mexico; msoto@colpos.mx

**Keywords:** nanobiotechnology, bacteria, phytopathogenic fungi, viruses, phytopathogenic nematodes, nanopesticides, nanofertilizers, nanosensors

## Abstract

Currently, the excessive use of pesticides has generated environmental pollution and harmful effects on human health. The controlled release of active ingredients through the use of nanomaterials (NMs) appears to reduce human exposure and ecosystem alteration. Although the use of NMs can offer an alternative to traditional methods of disease diagnosis and control, it is necessary to review the current approach to the application of these NMs. This review describes the most recent and significant advances in using NMs for diagnosing and treating plant diseases (bacteria, phytopathogenic fungi, viruses, and phytopathogenic nematodes) in cultivated plants. Most studies have focused on reducing, delaying, or eliminating bacteria, fungi, viruses, and nematodes in plants. Both metallic (including metal oxides) and organic nanoparticles (NPs) and composites are widely used in diagnosing and controlling plant diseases due to their biocompatibility and ease of synthesis. Few studies have been carried out with regard to carbon-based NPs due to their toxicity, so future studies should address the development of detection tools, ecological and economic impacts, and human health. The synergistic effect of NMs as fertilizers and pesticides opens new areas of knowledge on the mechanisms of action (plant–pathogen–NMs interaction), the interaction of NMs with nutrients, the effects on plant metabolism, and the traceability of NMs to implement sustainable approaches. More studies are needed involving in vivo models under international regulations to ensure their safety. There is still controversy in the release of NMs into the environment because they could threaten the stability and functioning of biological systems, so research in this area needs to be improved.

## 1. Introduction

Sustainable and eco-friendly agricultural crop technology must be improved to feed the anticipated 9.7 billion people worldwide by 2050 [1]. The 5000 known species of insect pests affecting agricultural crops [2] can cause losses of 10–25% [3] or 26–80% of crop production [4], which represents about USD 100 million annually [5,6,7,8]. In response to abiotic or biotic shock stress, plants quickly produce proteins and protective metabolites. Pests, diseases (due to viruses, bacteria, mycoplasma, fungi, and nematodes), and climatic shocks (drought, frost, high and low temperatures, hail, waterlogging) cause crop losses [9]. Plants defend themselves against stress through acquired resistance, genetic resistance, signal transduction, and pathogen-associated molecular pattern (PAMP)-triggered immunity [10]. After pathogenic infections, plants synthesize jasmonic acid, methyl jasmonate, and salicylic acid, among others [11]. The electrical and optical characteristics of nanoparticles may be used to manufacture materials for biosensing, detection [12], and control of phytonematodes, pests, and diseases.

Nanotechnology is the science responsible for designing, developing, and applying particles and devices at the scale of 1–100 nm, at which size the physical-chemical properties of the starting organic or inorganic materials change [13,14,15]. These extraordinary properties include shape, size, porosity, hydrophobicity, hydrophilicity, and large surface-to-volume ratios that increase the rate of chemical and biochemical activities [16,17]. Nanotechnology applied to biological and agricultural sciences is known as nanobiotechnology. Among the different types of nanoparticles (NPs), carbonaceous nanoparticles (CNPs) are the most widely used nanomaterials today due to their striking characteristics and various applications in diverse fields in agriculture [18].

Current agricultural applications of nanotechnology include several nanomaterials and nanodevices such as synthetic nanofertilizers and nanopesticides (bactericides, fungicides, nematicides, herbicides) that support the biological effectiveness of the active ingredients, and the use of alternative nanoformulations (i.e., essential oils, plant extracts, metabolites, and growth regulators), in addition to nanocarriers and nanosensors (disease diagnosis). They all promote agricultural and environmental sustainability and increase crop yields via growth promotion [19], soil improvement, crop protection, precision agriculture, stress tolerance, and plant breeding [20]. Nanoparticles (NPs) have antimicrobial characteristics which improve plant behavior and resistance to stress conditions [21]. NPs have a strong ability to transfer genetic components directly to the plant cells, functioning like transgenic vehicles of DNA [22]; nanoparticles as nanocarriers to introduce nucleic acids into plant cells for plant protection or genetic manipulation is an emerging area of research [22]. The mechanisms of uptake and translocation within the plant and the fate of nanoparticles and their cargo are almost unknown, so comprehensive research is required that takes into account environmental impact, biocompatibility, degradability and toxicity. Another challenge in this type of nanoparticles is the targeted delivery of genetic material (at the subcellular level) to alter metabolic pathways in specific organelles [23]. Recent studies of genetic engineering using nanomaterials as vehicles (delivery-targeted RNAi and CRISPR (Clustered Regularly Spaced Short Palindromic Repeats)/Cas: (CRISPR-associated protein) type and DNA sequences) are useful to continue conducting research in the genetic improvement of crops and the use of gene editing [1,24,25,26,27]. In contrast, nanotechnology can have potentially harmful effects that are still unpredictable on soil microorganisms, water, and ecosystems; the fate of some nanomaterials such as zinc and titanium oxides used as nanofertilizers that could create toxicity [28]; or novel green synthesized nanoparticles which could automatically be transferred to the next generation of plants (crops) via cytoplasmic maternal “inheritance type” if plants are regenerated from their in vitro cell culture. Thus, the possibility that NMs in the environment could put the stability and functioning of biological systems at risk continues to be a cause of concern in the scientific community. This review includes the recent nanomaterial advances for plant disease diagnosis and treatment with regard to bacteria, fungi, viruses, and nematodes.

With regard to the materials considered in this review, the most recent and relevant studies have been selected independently of the type of nanomaterial. Inorganic nanoparticles are the most commonly used in research and industry (metal 25% and metal oxides 54%), followed by those of carbon (10%) [29]. This is because metal nanoparticles can be easily synthesized and possess thermal, electrical, catalytic, and antimicrobial properties. Consequently, in the present review, studies involving metallic nanoparticles are mostly reported, followed, to a lesser extent, by other nanomaterials.

## 2. Classification, Characteristics, and Synthesis

**Figure 1:**
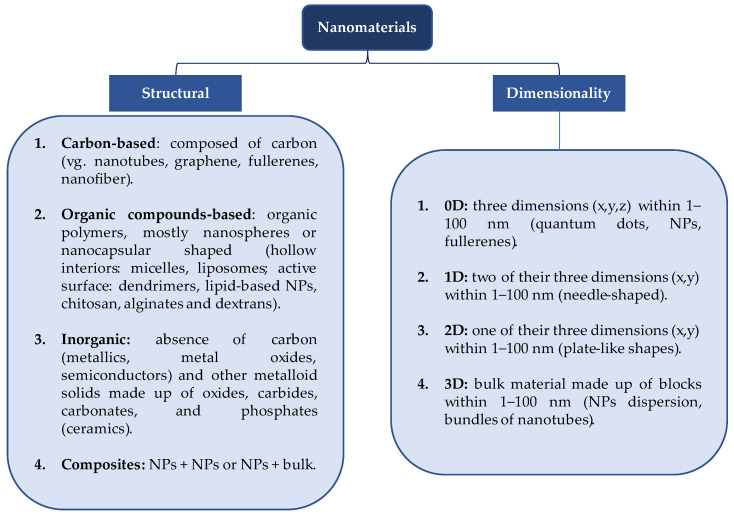
Classification of NMs according to their structure and dimensionality.

Although the terms “nanomaterial” and “nanoparticle” are sometimes used interchangeably, important differences in terms of their structure and dimensions have been reported in the scientific literature. Nanomaterials (NMs) have at least one of their dimensions in the range of 1–100 nm, while nanoparticles (NPs) have three dimensions in the nanoscale range [30]. Figure 1 shows the classification of NMs according to their structure/composition and dimensionality. As part of NMs, NPs have external dimensions in the range of 1–100 nm, and the lengths of the shortest and longest axis should not differ significantly according to the definition of the International Organization for Standardization (ISO/TC 229-Nanotechnologies [31]). If one of the dimensions changes significantly, the appropriate term should be used (e.g., nanofiber, nanowire, nanoplate). Thus, NPs are classified as inorganic, organic, and carbon-based. Inorganic NPs can be metallic (Al, Cu, Au, Ag, Fe, Zn), based on metal oxides (ZnO, TiO_2_, CuO, Fe_2_O_3_, F_3_O_4_, MgO; which gives them greater efficiency and reactivity compared to metallics), semiconductors (ZnO, Si, Mn), and ceramic (TiO_2_, carbides, carbonates, oxides) [32]. Organic NPs (Figure 1) are composed of organic compounds (lipids or polymeric) and include micelles, dendrimers, liposomes, and ferritin; they are non-toxic and biodegradable, and they have potential applications as nanopesticides (use of natural polysaccharides and proteins for encapsulation) [33].

On the other hand, dendrimers are often used for molecular recognition (nanosensors) and drug delivery, while lipid-based NPs are useful in agriculture as they are biodegradable and have a low level of toxicity [29]. Finally, carbon-based NPs are made entirely of carbon and mainly include fullerenes, carbon nanotubes, activated carbon, and graphene oxide. Their application as biochemical sensors in agriculture has generally been reported [34]. Table 1 describes the main categories of nanomaterials based on their structure, characteristics, and properties, synthesis method, and potential applications. As for carbon-based NMs, nanotubes have the disadvantage that mass production presents economic problems and reduced adsorption and desorption kinetics. Activated carbon is the first choice for manufacturing electrodes due to its low cost, high stability, and excellent surface area [35]. Graphene oxide requires the use of a large amount of water for purification purposes and can be dangerous because it tends to reduce thermally, causing the potential for explosion and damage to life, including a lack of stability that can have environmental consequences [36]. Unfortunately, carbon-based NMs are toxic to the environment and living things. Carbon nanotubes and fullerenes can cause damage to aquatic invertebrates, exhibiting cytotoxicity and altering phagocytosis, with characteristic features of macrophage necrosis and degeneration, lung inflammation, and lung cell proliferation in rats, as well as toxicity in mammalian cells [37]. Uncoated fullerenes are lipophilic and have an affinity for cell membranes; their redox activity can cause lipid peroxidation in bass brains and decrease total glutathione levels in gills; they also increase water clarity due to their bactericidal action [38]. Unlike carbon-based NMs, inorganic NMs are non-toxic, hydrophilic, biocompatible, and stable.

For this reason, the potential applications are biological, including drug administration, implants, and diagnostic techniques. They are obtained by dividing pieces of bulk material (top-down approach) or by integrating individual atoms and molecules into large nanostructures (bottom-up approach) [43]. In the bottom-up approach, there is greater control in the synthesis procedure. However, the methods are more expensive and less scalable; they involve chemical methods (chemical reduction, bioreduction or biological synthesis, sol-gel synthesis, thermal decomposition, ultrasound, microwave) and laser ablation [44]. Biological synthesis uses natural sources (plant extracts or in vivo) such as microorganisms and plants for the production of nanomaterials; in the case of green synthesis (plants), organic molecules such as phenolic compounds (flavonoids and tannins), terpenoids, and proteins act as reducing agents for the reduction of metal ions and as stabilizers that surround the nanomaterials to stop their agglomeration and provide long-term stability [45]. The top-down approach involves mechanical/physical methods such as milling (limited control of shape and size), laser ablation, chemical etching, electrochemical oxidation, arc discharge and sonication, and sometimes also chemical methods (nanolithography), especially for manufacturing semiconductor devices (integrated circuits) [43,44].

## 3. Application for Plant Disease Diagnosis

Nanostructures have great potential with regard to fabricating biosensors. Nanosensors are electronic devices with two parts: sensing and electronic data processing [46]. The sensing part is extremely useful because it can sense heat, light, pH, temperature, drought, pathogens, humidity (water), plant-emitted chemical compounds, fertilizers, contaminants, pesticides, herbicides, microbial toxins, metabolites, DNA, proteins, enzymes, antibodies, and nutrients in real time. Sensors can recognize and detect living cells, tissue, bacteria, viruses, fungi, and specific chemical substances (salicylic acid) [13,47,48,49,50]. Devices are gadgets based on nanosensors that detect infections in plants/seeds and are available for some plant pathogens [51]. Nanosensors make possible the rapid quantification and detection of bacteria, fungi, viruses, pathogens, and toxins present in crops, soils, and foods, thereby reducing yield losses and increasing biosafety.

**Figure 2:**
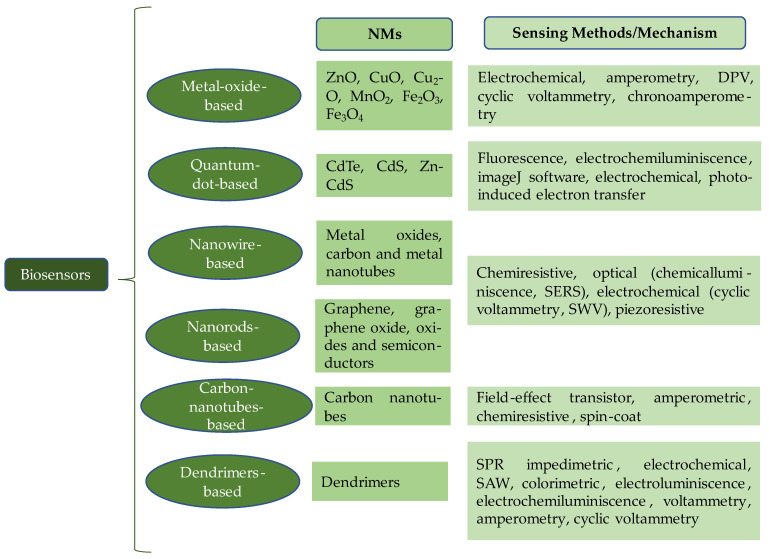
The different nanomaterials-based biosensors and their sensing methods/mechanism. DPV: Differential Pulse Voltammetry; SERS: Surface-enhanced Raman Scattering; SWV: Square Wave Voltammetry; SAW: Surface Acoustic Wave.

Biosensors based on NMs for the diagnosis of plant diseases must be selective, reproducible, stable, and sensitive; these characteristics depend on specific criteria such as the type of bioreceptor and the type of transducer used to prepare the device, as well as the technology and type of detection system used [52,53]. Figure 2 classifies biosensors based on the NMs used for their manufacture, and the diagnostic and/or detection methods. Metal oxides, mainly zinc, copper, iron, and manganese, are widely used to create electrochemical biosensors, which take advantage of their magnetic and semiconductor properties and the high speed of electron movement [54]. In the case of ZnO, the various dimensions of the nanostructures offer multiple applications. Those of dimension 0 (0D) have a high surface area for immobilizing molecules, which facilitates the interaction between target analyte and sensor elements (leading to higher sensitivity); 1D structures are elongated and facilitate the transport of electrons (leading to greater precision), while 2D structures offer immobilization planes that allow various analytes to be detected; and 3D structures have additional surfaces for immobilization (leading to higher sensitivity and efficiency) [52]. Furthermore, with regard to the design of biosensors based on NMs, the use of various types of NMs for their manufacture is common. For example, ZnO and carbon nanotubes can act as a tool to investigate the electrochemical oxidation of naproxen and its voltammetric determination [55].

Depending on the transducers used, biosensors can be piezoelectric, calorimetric, thermoelectric, pyroelectric, magnetic, electronic, optical, or electrochemical. The most common detection methods in NMs-based biosensors involve different mechanisms (Figure 2). In the case of electrochemical biosensors, for example, an electrical signal dependent on conductance, resistance, and capacitance is produced on the surface of the biosensor, proportional to the concentration of the analyte [56]. These are generally used to detect pathogens and are highly sensitive as there is no interference from fluorescent compounds or absorbents. Electrochemical biosensors are classified as voltammetric (detecting the analyte according to the change in applied current and potential), amperometric (measuring the current generated through electrooxidation/reduction in the biological recognition reaction on the electrode surface), and potentiometric (absorbing an ionic species during the reaction that is detected by an ion-selective electrode) [56]. Another type of biosensor is optical, in which an optical transducer is used, and the analyte is detected using light; the most common are fluorescence and luminescence (photodiodes or photomultipliers detect light) and surface plasmon resonance (taking advantage of the surface charge density oscillations of free electrons at the metal–dielectric interface) [57]. Finally, in the case of piezoelectric biosensors, a crystalline and mechanical oscillation occurs (affinity interactions); in this case, the piezoelectric platform works according to the change in oscillations due to a mass attached to the surface, such that the surface can be modified with antibodies or antigens, polymers with genetic information such as DNA, or receptors of organic origin [58]. Piezoelectric transducers generally have a quartz crystal microbalance (a circular square of quartz with metal electrodes on opposite sides). Thus, the thickness of the plate defines the resonance frequency, and is widely used to determine the presence of viruses, bacteria, and small molecules such as pesticides [59]. Both piezoelectric and electrochemical biosensors are analytical devices that can contain an antibody as a biorecognition element, such that the specificity of the antibody influences the specificity of the immunosensor. In this case, immunosensors contain immobilized antibodies with the ability to recognize antigens, although the opposite reaction is also possible in that nanoparticles, carbonaceous materials, quantum dots, enzymes, and fluorescent tags are used to increase the sensitivity and specificity of immunosensors for various types of analytes, with electrochemical, optical, piezoelectric, and colorimetric detection schemes [58,60].

### 3.1. Bacteria

Nanomaterials have also been used in biosensor design; in this case, they can go together with biomolecules that will recognize the bacteria of interest, or be part of the transducer system. The recognition biomolecule and transducer work together to produce a measurable signal that the final user can visualize. Platforms used as probes for bacterial detection usually depend on fluorescence or colorimetric signal readouts. Some nanomaterials used to detect bacteria with the use of a biosensor are based on carbon, such as graphene and quantum dots, gold nanomaterials, silver nanomaterials, magnetic nanomaterials, semiconductor nanomaterials such as copper oxide (CuO), zinc oxide (ZnO), zinc peroxide (ZnO_2_), silicon dioxide (SiO_2_), and titanium dioxide (TiO_2_), amongst others. The nanomaterials act as unique sensing platforms for probing interactions between them and the target bacteria, because nanomaterials have a high surface-to-volume ratio, possess the possibility of modulating their shape, size, arrangement, and composition, and have the ability to modify surfaces with a wide range of molecular ligands. These characteristics increase the sensitivity, selectivity, and the conductive, thermal, and optical properties of the biosensor, as has been demonstrated in various works [61,62]. For instance, Sealy [63] developed an electrochemical biosensor based on TiO_2_ nanorods from 80 to 100 nm in dimension which were adhered to two electrodes to detect *Escherichia coli*. Bacteria detection occurs when a synthetic analog of a natural protein enzyme (DNAzyme) previously adhered to the electrode encounters a specific bacterium; then, a fragment of the DNAzyme is detached and immobilized on the opposite TiO_2_ electrode. Consequently, the breaking of the TiO_2_ heterostructures on one electrode and its re-building on the other changes the electrical current measured in the device. Others work as shown in Table 2.

### 3.2. Phytopathogenic Fungi

Plants produce signals in response to attack by phytopathogens such as enzymes, gaseous molecules, and secretory compounds. These can be used as biomarkers for the development of nanobiosensor platforms [67,68].

The nanotechnological tools recently used for the diagnosis of diseases caused by phytopathogens are quantum dots (QD), nanodiagnostic kit, microneedle patches, nanopore sequencing, nanobiosensors, nano barcoding, metal nanoparticles, miRNA-based nano diagnosis, and array-based nanosensors [69]. In the case of nanotechnologies used for the diagnosis of phytopathogenic fungi, there are reports on the use of nanopore sequencing for the diagnosis of *Colletotrichum* sp., *Fusarium oxysporum* f. sp. *lycopersici*, *Verticillium dahlia*, and *Penicillium digitatum* [70].

Although there are reports on the use of nanomaterials for the diagnosis of plant pathogens, for example, through the use of enzyme-based biosensors coated with Au, Ag, Cu, or Ti-NPs [71], in the case of phytopathogenic fungi such nanomaterials are still in innovation, and more research is required in this regard (Table 3).

### 3.3. Viruses

In cultivated plants, around 50% of the incipient biotic diseases are caused by viruses. Consequently, novel and efficient technologies and strategies are required to allow timely diagnosis and management to avoid productivity losses in global food production. In general, symptoms in virus-infected plants take the form of decreasing growth, chlorosis, deformation, and mottling [78]. The treatment of phytoviruses is complex due to their genomic diversity and evolution, as well as the presence of insect vectors, so alternative management options to the use of pesticides are needed to guarantee effectiveness without consequences for the environment [79,80]. When nanotechnology is applied to detect, diagnose, or treat viruses that cause diseases in cultivated plants and/or are of importance to humans, we refer to this as nanophytovirology. Plant viruses can be used for the development of nanovehicles (nanomaterials conjugated with biocompatible molecules) with applications in the medical and agricultural fields for the delivery of active molecules [81]. As we will see later, studies with regard to using NPs to control viral diseases in plants are limited in number, particularly with regard to crops and direct applications in the field. Nanophytovirology as an emerging science is in an incipient stage that requires multidisciplinary research focused on the study of the interaction of nanomaterials with plant nutrition, the vector–virus–NPs relationship, and the activation of antiviral defense mechanisms in plants [78]. The most recent studies on the use of NPs for diagnosing viral diseases in plants are shown in Table 4 described below.

The development of sensors for detecting and diagnosing viruses in plants is possible thanks to the unique properties of nanoparticles. In the case of metal nanoparticles, the phenomenon of surface plasmon resonance allows the electrons in the surface layer of metals to be excited by light photons with a certain angle of incidence, so that these electrons can propagate on the surface of the metal. Any change in the refractive index or resonance angle/frequency then allows biological recognition, enabling the development of sensors [56]. Nanostructured materials can be conjugated with biological systems that provide specificity and selectivity to the system. When antibodies are used, immunosensors are produced which allow the detection of viral antigens [78]. Biological recognition receptors are immobilized on analytical surfaces to interact with specific analytes and produce physical or chemical changes that are ultimately converted to an electrical or optical signal [87]. Nanostructured materials serve as surfaces or analytes. Metal (Au) and carbon (C) nanoparticles, quantum dots, and minerals (quartz) have been used in virus biosensors in plants [82,83,84,85]. The physical and chemical phenomena produced by the biosensor–virus interaction involve changes in mass and resonance frequencies, impedance and electrochemical resistance, conductance, and fluorescence (Table 4).

In developed countries, plant-parasitic nematodes reduce crops by 8.8%, and in tropical and subtropical regions of the world by 14.6%. Root-knot nematodes alone have more than 3000 host plant species, both cultivated and wild [7].

### 3.4. Nanotechnology in Nematodes Diagnosis

Gadgets based on the use of nanosensors to detect infections in plants/seeds have become available for some plant pathogens [51]. There are a number of advanced studies with regard to nanoparticles and biosensors for detecting and recognizing nematodes and their infections, but there are not commercially available complete, fast, costless protocols with nanosensors for direct and specific nematode diagnosis and their infections. However, detecting nematodes quickly and directly will soon be possible.

The parasitic nematode *Caenorhabditis elegans* has been detected using pH-sensitive radiometric nanosensors (40 nm) [88]. *C. elegans*, a plant pathogenic nematode, was used as a biosensor model for treatments involving single-walled carbon nanotubes (SWCNs) [89]. DNA has been used as a biosensor (nanochips) to detect nematodes, eggs, infested tissue, soil samples after infection, and any abnormal changes in soil fauna [90]. Susic et al. [91] used DNA to detect nematodes in potato tuber tissue. Zhou et al. [92] have used DNA as a biosensor for rapidly detecting nematodes in potato tubers and the pine wood nematode *Bursaphelenchus xylophilus* (PWN), which can reduce nematode damage. Nanochips are being prepared in DNA sensors. Nanochips are microarrays that include fluorescent oligo-capture probes to detect the extent of DNA hybridization. They are highly sensitive and can identify point mutations (single nucleotide changes) in viruses and bacteria [93]. The PCR method has been proposed as easier, faster, more specific, and relatively costless when it comes to diagnosing the presence of nematode species in soil using commercial kits incorporating collected soil samples.

## 4. Application for Control of Plant Diseases

### 4.1. Bacteria

Bacteria colonize and multiply rapidly in the tissues of living plants, quantifying millions of microorganisms per tissue area in a short time. Their growth and the production/release of bioactive compounds that directly interfere with biochemical signaling pathways and host physiology cause excessive drain of nutrients and alteration in appearance (disease symptoms), plant development, and crop yield. The damage to crops as a result of bacterial diseases may be minor compared to those caused by other phytopathogenic microorganisms, but it impacts local, regional, and global agricultural production [94].

According to Carezzano et al. [95], the main Gram-negative phytopathogenic bacteria that colonize plant tissues are grouped as (1) bacteria that colonize xylem vessels: *Erwinia* spp., *Pectobacterium carovotum*, *Xylella fastidiosa*, *Xanthomonas campestris*, *Pantoea stewartii*, *Clavibacter michiganensis*, and *Ralstonia solanacearum*, (2) bacteria that colonize root tissues: *Rhizobium radiobacter* and *Agrobacterium rhizogenes*, (3) bacteria that colonize parenchymal tissues: *Pseudomonas* spp., *Pseudomonas syringae*, and *Acidovorax citrulli*, amongst others. In addition, there is a phloem-limited Gram-negative bacteria *Candidatus* Liberibacter solanacearum belonging to the family Rhizobeaceae [96]. In contrast, the principal genera of Gram-positive phytopathogenic bacteria are *Arthrobacter*, *Clavibacter*, *Curtobacterium*, and *Rhodococcus* [97]. These bacteria have a broad host range, infecting herbaceous and woody plant species. Hence, diagnosing and treating plant diseases caused by phytopathogenic bacteria in fields, greenhouses, and food storage facilities are essential to maintaining food security and maximizing agricultural productivity and sustainability [98].

Bacteria are currently hard to handle for reasons such as the ever-changing climate, inappropriate cultivation methods, high resistance to typical treatments, and only small numbers of agrochemicals able to target them. Specifically, they are controlled by bactericides that may be of a chemical and biological nature such as phenylmercuric acetate, chlorothalonil, chlorine dioxide, quaternary ammonia compounds, some commercial products such as saijunmao, kocide, thiodiazole copper, zhongshengmycin and bismerthiazol, as well as irradiation from a cobalt-60 source, *B. thuringiensis* products, neem, essential oils, plant extracts, bacteriophages, and nanobactericides, amongst others. Specifically, nanobactericides could be referred to as engineered nanostructure compounds used for bacteria treatment. These nanoforms release active ingredients in a controlled form for long periods with a slow degradation rate, making them particularly efficient in controlling plant diseases. The nanobactericides are considered relatively less toxic, environmentally safer, and more eco-friendly than conventional bactericides. They can be found in the form of nanogels, nanoemulsions, nanocapsulated formulations based on metal oxide, and metal nanoparticles, as reported by Kumar et al. [99] and Wang et al. [100]. The action mechanisms associated with the biocidal activity of nanomaterials on bacteria depend on the size of the nanomaterial and the type of material used. For example, silver nanoparticles (Ag-NPs) inhibit ATP production and DNA replication, damage the bacterial cell membrane directly, increase oxidative stress, and subsequently, lead to cell death. In contrast, zinc oxide nanoparticles (ZnO-NPs) stimulate the production of reactive oxygen species and the release of zinc ions (Zn^2+^). Finally, copper oxide nanoparticles (Cu-NPs) damage the bacterial cell membrane, go into cells, and modify their enzymatic function, all of which lead to their death [101].

Some examples of the use of nanomaterials to control bacteria growth in plants were reported by Fan et al. [102], who recommended spraying 0.25 mg mL^−1^ of ZnO-NPs on tobacco (*Nicotiana tabacum*) leaves to control *P. syringae*. They observed that ZnO-NPs were able to inhibit the pathway of biofilm formation and increase the activity of enzymes such as superoxide dismutase (SOD), peroxidase (POD), and catalase (CAT). In another case, García-Sánchez et al. [103] formulated nanospheres based on cationic poly (*N*,*N*-dimethyl aminoethyl methacrylate) di-block copolymers of 50 nm in size to combat *Candidatus* Liberibacter solanacearum in tomato plants (*Solanum lycopersicum*). They observed in the plant a reduction in typical symptoms produced by bacteria and a low presence of bacterial DNA after 25 days using 70 ppm of these nanospheres. Other reports on the use of nanomaterials as bacterial treatments are described in Table 5.

### 4.2. Phytopathogenic Fungi

Agricultural losses due to plant pathogens are estimated at 20% to 30% per year. For this reason, the diagnosis and management of crop diseases are essential. Strategies to reduce losses have focused mainly on agrochemicals. However, their intensive and indiscriminate use has harmed the environment [69,110]. Given this scenario, the need arises to generate alternatives for managing plant pathogens and promoting sustainable agriculture. Nanoparticles (NPs) have aroused great interest in agriculture due to their antimicrobial activity and are considered an alternative when it comes to diagnosing and managing plant diseases [111,112]. Their potential to reduce the incidence and severity of phytopathogenic fungi has been reported, and they also favor plant growth [113,114]. In particular, metal oxide nanoparticles (Zn, Cu, Fe, Mg, Ti) are considered an efficient and ecological alternative for controlling phytopathogenic fungi in agriculture [115]. Ag, Cu, Fe, Zn, and Ni nanoparticles have been shown to have excellent antifungal properties in in vitro evaluations [115,116]. These results provide a promising scenario for a new generation of fungicides [112]. In this sense, recent research has evaluated the antifungal effect of nanoparticles under in vivo conditions, which provides a broader overview of their applicability in food production.

Ag nanoparticles. In a study carried out in Pakistan by Ansari et al. [113], silver nanoparticles (AgNPs) were obtained through green synthesis from leaf extracts of the neem tree. The AgNPs were evaluated to determine their potential against the early blight of tomato (*Alternaria solani*). Six concentrations of AgNPs were applied to the leaves of tomato plants (*S. lycopersicum*) every 15 days until harvest. The results indicated that the tomato plants treated with AgNPs significantly reduced the disease severity index (73%) and incidence (69%) compared to control plants. In addition, the treatment led to a significant increase in plant weight (30%), number of leaves, fresh weight (45%), dry weight (40%), chlorophyll, alkaloids, total soluble sugars, flavonoids, and total soluble protein in the tomato plants, compared to control plants.

Cu nanoparticles. Copper oxide nanoparticles (Cu_2_ONPs), manufactured by chemical methods, were evaluated in Egypt to determine their effect on root rot disease in cucumber caused by *Fusarium solani*. Cu_2_ONPs were applied at 0.30 and 0.35 M. The results showed that 0.30 and 0.35 M of Cu2ONPs significantly reduced the incidence and severity of the disease. The analysis with the use of a scanning electron microscope showed that the mycelium and the spores of *F. solani* treated with Cu_2_ONPs suffered alterations such as twisting, plasmolysis, and collapsing. At the same time, the plants treated with the nanoparticles developed thicker cell walls, root cortex, and mesophyll tissue. For this reason, the growth and yield of the cucumber plants improved when treated with Cu_2_ONPs [114].

In another study, also carried out in Egypt, the antifungal activity of copper nanoparticles (Cu-NPs) was evaluated against postharvest diseases caused by *Botrytis cinerea* and *S. sclerotiorum* in cucumber (*Cucumis sativus* var. Hesham F1 hybrid). The nanoparticles were obtained by green synthesis using copper sulfate as a precursor in the presence of polyvinylpyrrolidone (PVP) as a stabilizer, and ascorbic acid was used to reduce CuSO_4_ to Cu-NP. In this study, cucumber fruits were inoculated with both phytopathogenic fungi, for which they were immersed in colloidal Cu-NP solutions (50 and 100 µg/mL). The antifungal activity of the NPs improved as their concentration increased, and the severity of postharvest diseases was reduced [117].

Fe nanoparticles. In another study, also with regard to cucumber cultivation, the antifungal potential of Fe_2_O_3_ conjugated with boron (Fe_2_O_3_ NP-B) and Fe_2_O_3_ conjugated with humic acid (Fe_2_O_3_ NP-H) was evaluated against *F. oxysporum*, which causes cucumber wilting. The nanoparticles were synthesized using gamma rays. The application of Fe_2_O_3_ NP-B (0.25 mM) and Fe2O3 NP-H (0.125 mM) reduced the incidence of the disease. The best treatment was Fe_2_O_3_ NP-B, which reduced disease rates (20.83%) and provided high protection against disease, followed by Fe_2_O_3_ NPs-H (25%). Additionally, Fe_2_O_3_ NP-H and Fe_2_O_3_ NP-B improved the plant’s morphological traits, such as photosynthetic pigments, total phenols, and the activity of antioxidant enzymes [118].

Zn nanoparticles. In a study carried out in Egypt, zinc nanoparticles were obtained by myco-synthesis using a filtrate from *Aspergillus fumigatus* OQ519856. The effect of ZnO NPs CuO NPs and ZnO-CuO NPs against *Fusarium* sp. was evaluated in the broad bean (*Vicia faba*) CV Giza 3 variety. The seeds were placed in immersion in a nanosolution of 125 μg/mL for 2 h. Subsequently, they were placed in pots (one seed per pot). After germination and the appearance of cotyledons, the nanosolutions were applied weekly at a concentration of 125 g/mL. The results indicated that ZnO-CuO NPs decreased the disease index (22.5%) and increased protection (74.28%). In addition, plant growth, photosynthetic pigments, carbohydrate and protein content were improved, and the number of pods per plant (146.1%) and pod weight (228.8%) increased compared to control plants [119].

Ni nanoparticles. In a study carried out in India, the antifungal activity of Ni_0.5_Al_0.5_Fe_2_O_4_ nanoparticles against *F. oxysporum*, which causes dry rot in ginger, was evaluated. The synthesis of the nanoparticles was carried out using the hydrothermal method. The rhizomes of ginger (Suprabha variety) were treated with different concentrations of Ni_0.5_Al_0.5_Fe_2_O_4_ (0.1 mg/mL, 0.2 mg/mL, 0.3 mg/mL, 0.4 mg/mL, and 0.5 mg/mL). Ginger rhizomes were immersed for 45 min in solutions with different concentrations of Ni_0.5_Al_0.5_Fe_2_O_4_. Subsequently, they were planted in pots and placed in a polyhouse. The disease incidence decreased as the concentration of nanoparticles increased. In the case of Ni_0.5_Al_0.5_Fe_2_O_4_ nanoparticles, a 500 ppm concentration of nanoparticles reduced the disease incidence (22.8%) compared to control plants (72.3%) [112].

S nanoparticles. In another study also carried out in Egypt, the antifungal activity of sulfur nanoparticles (S-NPs) was evaluated against postharvest diseases caused by *B. cinerea* and *S. sclerotiorum* in cucumber (*C. sativus* var. Hesham F1 hybrid). The nanoparticles were obtained from sodium thiosulfate. The cucumbers were immersed in a colloidal solution of S-NPs (25 and 50 μg/mL). The application of S-NPs reduced the disease incidence, both at a concentration of 25 μg/mL (75%) and at a concentration of 50 μg/mL (50%) compared to control plants that reached 100% of disease incidence [117]. The main contributions of these investigations are summarized in Table 6.

### 4.3. Viruses

The most recent studies on the use of NPs for controlling viral diseases in plants are shown in Table 7 described below. AgNPs have been one of the most frequently used tools to control virus infection in plants, although the use of Zn, SiO_2_ and Fe_3_O_2_, CeO_2_, TiO_2,_ and Au nanoparticles has also been reported. Those synthesized chemically and biologically (from microorganisms and plants) have positively affected the induction of systemic resistance and improvements in plant defense. However, many researchers have shown that biosynthesis is more environmentally friendly, more affordable, and less risky compared to chemical synthesis. Studies have shown that post-infection application of NPs (24 h to 3 d, *V. faba*, *Chenopodium amaranticolor*, *Solanum tuberosum*, *Lactuca sativa*) significantly reduces symptoms, infection, and virus quantity, improving the biochemical processes and defense systems involved (photosynthetic process, enzymatic activity, and phytohormone synthesis). In addition, fewer modifications in morphology and tissue structure have been reported (Table 7). However, the application of NPs pre-infection has also shown positive effects by reducing the severity of the diseases in infected plants due to the expression of genes associated with signaling molecules (e.g., salicylic acid and jasmonic acid) for the activation of defense mechanisms [120]. Cai et al. [121] showed that the application of NPs pre-infection with the tobacco mosaic virus increased the production of reactive oxygen species (ROS) and the activity of antioxidant enzymes (catalase-peroxidase), the expression of genes associated with systemic resistance, and an increase in abscisic and salicylic acid levels. According to Dutta et al. [78], the use of metallic NPs in plants to control viruses produces changes in the balance of growth regulators, such as the increase in the biosynthesis of cytokinins, abscisic acid, and brassinosteroids, which leads to the activation of the antioxidant defense mechanism of the plant. The use in chili plants of TiO_2_ NPs up to 6 and 8 h before infection can decrease viral concentration, and reduce the symptoms caused by the tobacco mosaic virus [122].

The induction of biotic and abiotic stress, depending on the concentration of NPs, can trigger the synthesis of secondary metabolites such as phenolic compounds, flavonoids, anthocyanins, and glucosinolates [78]. Carbon-based nanomaterials have also produced morphological, physiological, and molecular changes in virus-infected plants. Al-Zaban et al. [131] showed that C60 fullerenes applied foliarly for 21 d suppressed cucurbit chlorotic yellows virus infection in *N. benthamiana* up to 5 d after inoculation, while nanoscale Fe and Zn did not suppress viral progression. With C60 fullerenes, gene transcripts were reduced five-fold, and defense phytohormones (abscisic acid and salicylic acid) increased by more than 40%, producing a dual effect in the form of viral inhibition and protection.

Improvements have been observed in the growth and development of plants treated with NPs (in terms of an increase in fresh and dry matter) and in the yield, chemical composition, secondary metabolites, and quality variables in fruits and tubers. In plants, NPs interact with the glycoproteins of the virus envelope, causing fracture patterns that interfere with the recognition of the virus by the host cell; AgNPs can also adhere to the capsid protein and interfere with the replication process [78,129,130].

A promising area in controlling viral diseases in plants is using nanomaterials as carriers of antiviral molecules. Mitter et al. [132] applied RNA (pathogen-specific double-stranded RNA) loaded on layered double hydroxide clay nanosheets. RNA was released in a controlled manner up to 30 d after application, providing protection against cucumber mosaic virus and pepper mild mottle virus on sprayed leaves and newly emerged unsprayed leaves. Furthermore, bilayer lipid vesicles known as nanoliposomes can also be used to encapsulate and deliver bioactive ingredients. In this case, Wang et al. [133] showed greater efficiency in controlling viral diseases in the field when using 117 nm quercetin nanoliposomes compared to the use of conventional quercetin.

### 4.4. Nanoparticles in Nematode Control

The sedentary endoparasites *Meloidogyne* sp., *Heterodera* sp., and *Globodera* sp. are the most crop-damaging nematodes because they affect many important crops, from grasses to trees [5]. Host resistance is widely regarded as an eco-friendly and economically viable alternative to chemical treatments. Many R-genes (resistance genes) have been isolated and characterized, especially from wild hosts, which confer resistance primarily against sedentary endoparasitic nematodes [134,135]. Drug carriers with a controllable target nematicide and releasing system are recent nanotechnology products used for nematode control [13,136]. Carbonaceous nanoparticles (CNPs) are the most widely used today with regard to nematode control due to their striking characteristics and various agricultural applications [18]. Coated copper nanoparticles with polyethylene glycol 8000 and copper-doped zinc oxide nanoparticles with diethylene glycol were synthesized and studied as nano-fungicides and nano-nematicides; overall, it was found that foliar application of Cu- and Zn-synthesized nanoparticles could be a promising tool for controlling phytopathogenic fungi and nematodes [137]. The plant pathogenic nematode, *C. elegans*, is used as an experimental model to prove the activity of different nanoparticles types against phytonematodes, and predicting the potential applications of NPs with regard to a range of different aspects [138].

Green-synthesized nanoparticles are novel as nematicides [139]. The green biosynthesis of silver nanoparticles (Ag-NPs) using an aqueous extract of *Ficus sycomorus* leaves (FS-Ag-NPs) with nematocidal activity was first reported by Elkobrosy et al. [140] as a recommended treatment for managing plant-parasitic nematodes due to its simplicity, stability, cost-effectiveness, and environmentally safe nature. Considerable research is being undertaken to synthesize silver nanoparticles using plant extracts as the biological base in order to ensure “clean”, “nontoxic”, “harmless”, and “eco-friendly green chemistry”. Nowadays, silver NPs (AgNPs) are the most frequently utilized form of nanoparticles that have emerged as a superior product for controlling phytonematodes because they possess sufficient conductivity, have an excellent catalytic attribute, and are chemically stable [141]. As nematicides, plant-based AgNPs have revealed a very significant control of *Meloidogyne incognita* by reducing the number of galls and egg mass and improving tomatoes’ growth and fresh weight [141,142]. Among other synthetic types, metal nanoparticles used as phytonematicides are gold, platinum, TiO_2_, selenium (Se), and zinc (Zn) [143,144,145,146]. Although it can have ecotoxicity [147,148,149], copper (Cu) can also be applied to nematode control. Overall, foliar-applied Cu and Cu-doped ZnO NPs could be promising tools to control phytopathogenic fungi and nematodes, contributing to the sustainability of the agri-food sector. Another emerging area is the development of nanovehicles designed to deal with non-pathogenic plant viruses and bacteriophages, which are used to encapsulate active ingredients such as nematicides. Although most applications are for medical purposes, these nanovehicles are more advantageous than synthetic nanoparticles due to their biocompatibility and biodegradability [81].

## 5. Impact on the Environment and Human Health

NMs have potential applications in the agricultural sector in the form of nanofertilizers, water filtration systems, food packaging, pesticide development, biosensors, genetic manipulation, soil and waste management, and ensuring “sustainable” food production. Although we have been consuming NMs for years—some naturally produced and others involuntarily produced (anthropogenic NMs)—the increase in the “intentional” production of NMs (designed NMs) and their growing applications (both biological and industrial) have generated problems of toxicity and contamination [150]. According to Kumah et al. [151], metallic NPs (zinc oxide, silicon dioxide, titanium dioxide, and silver) and those based on carbon (nanotubes) are highly toxic because they produce cell death, oxidative stress, DNA damage, apoptosis, and induction of anti-inflammatory responses, both in immortalized cell lines and primary cells as biomarkers (both isolated from animal tissues). The problem is that immortalized cell lines do not reflect the mechanism observed in their normal homologous cells, primary cells are limited in quantity, and there are usually variations between batches. The use of NPs is a reality in today’s world, with commercial applications in textiles, water treatment, environmental remediation, cancer therapy, radiology, cosmetics, and food production. TiO_2_ NPs, for example, are widely used in biomedicine, although they can be released from the surface of titanium implants, enter the systemic circulation, and migrate to the brain [152]. Environmental pollution and consumer products containing NPs can cause accumulation in the human body, mainly in the brain, causing neurotoxicity [153]. Unfortunately, most toxicity studies are performed in in vitro models to avoid the use of animals. For example, Mancuso and Cao et al. [154] used human bone marrow mesenchymal stem cells to evaluate the cytotoxicity of CuO NPs, showing significant particle size-dependent effects, showing that smaller NPs are much more toxic than larger ones. Table 8 describes selected studies on some of the effects that NMs have on human health and the environment. The most serious problem after exposure to NMs and NPs is accumulation, since it is known that the generation of reactive oxygen species and the accumulation of autophagosomes (observed in in vitro studies) is related to the appearance and progression of neurodegenerative diseases [153]. Although uncontrolled exposure to NMs produces toxicity, the selective induction of cytotoxicity in cancer cells could help the safe development of anticancer therapies [155]. In addition to size, properties such as isotropy or anisotropy significantly influence the effect of NMs on cellular toxicity.

Regarding the effect of NMs in the environment, cytotoxic, genotoxic, and epigenetic effects have been reported in plants and other components of the environment, such as the soil microbiome (nitrifying bacteria) and marine organisms (zebrafish) [157,158,159,160]. Biochemical and physiological changes, alterations in the composition of photosynthetic pigments and photosystems have been reported in algae and aquatic plants dependent on light schemes (illumination before or after exposure to NPs) [161], impacts on carbon fixation, and water use efficiency during photosynthesis by exposure to CeO_2_ NPs [162]. In *Spirodela polyrhiza*, for example, exposure to AgNPs caused damage to the photosynthetic process and oxidative stress [163], with the toxicity mechanisms being related to the inhibition of the maximum and effective quantum yield of photosystem II, changes in fluorescence quenching, production of ROS in chloroplast, and inhibition of the activity of ribulose-1, 5-bisphosphate carboxylase-oxygenase (RUBISCO). Apparently, intracellular AgNPs dissociate into Ag+ ions, which are highly toxic. The emission of NMs to the environment (naked, coated, chemically or physically transformed) can occur through release during production, during use, or after disposal of products containing NPs, either directly or indirectly (plant effluents, wastewater treatment or landfills), with the most relevant NPs by production volumes being those made of TiO_2_ and SiO_2_, CeO_2_, FeO, AlO, ZnO, and carbon nanotubes [164]. For example, CeO_2_ NPs can stimulate growth and improve the rate of photosynthesis (higher RUBISCO activity) by up to 54% in soybeans when used naked than when coated with polyvinylpyrrolidone (36%) [162]. According to Moore [165], NPs can have harmful effects on biota (aquatic and terrestrial ecosystems) through the formation of ROS (damage to biological structures), release of ions, internalization, and the coating of biological surfaces.

## 6. Conclusions and Perspectives

For bacteria, there are many works focused on reducing, retarding, and eliminating their growth in plants using metallic nanomaterials, and a few studies have been directed at developing detection tools, which is an opportunity for new research. In vitro experimentation has allowed the development of a new generation of NPs-based fungicides and disease diagnosis techniques incorporating nanotechnologies. More recently, research has been conducted in fields, greenhouses, and nursery conditions, evaluating the antifungal activity of nanoparticles to reduce the incidence and severity of plant diseases, while acting as nanofertilizers to improve plant growth, development, and health. Regarding viruses, both metallic- and carbon-based NPs present a new strategy for viruses’ control in plants; however, more research is needed on the interaction between vectors–virus–plant NPs. Unfortunately, the use of nanoformulated nematicides with synthetic active ingredients still predominates, so the development of nanonematicides formulated with active ingredients from plant origins and friendly to the environment is expected. There are not commercially available complete fast and costless protocols with nanosensors for direct and specific nematode diagnosis.

Multidisciplinary studies are required incorporating a comprehensive approach that considers integrated disease management, plant metabolism, optimal doses, residue behavior, ecological impact (soil, water, fauna, etc.), economic impact, accumulation and cytotoxicity, the effects on humans, and the consequent acceptance on the part of agricultural producers. Many studies related to toxicity are contradictory and inconsistent, which is why in vivo toxicological models subject to international regulation are needed to ensure the safety of NPs.

## Figures and Tables

**Table 1:** Categories of principal NMs and their characteristics/properties, applications, and synthesis method [39,40,41,42].

NMs	Characteristics/Properties	Applications	Synthesis
Carbon-based Made entirely of carbon			
Nanotubes	Cylindrical and long tubular structure; single-walled or multi-walled; diffusion rate and electron kinetics of the reaction enhancing; compact size, high sensitivity, high specific surface area, and aspect ratio; point charges in the surface	Catalysis, chemical sensors, hydrogen storage systems, wastewater treatment, elimination of bacteria and viruses from water	Arch discharge method, laser ablation, chemical vapor deposition
Fullerenes	C60 is the most significant, cage-like structure; electron acceptor, high surface area	Electrode material, electrochemical biosensors, catalysis, fuel cell electrodes, magnetic resonance imaging, oxidation reduction reactions, stabilization of immune effect cells.	Arch discharge, detonation
Activated carbon	Or charcoal; tiny, low-volume pores, adsorbent, removal of foulants	Water purification processes (heavy metals, dyes, and gases); removal of contaminants from wastewater; removal of pharmaceutical and personal care products, organic/inorganic pollutants, pesticides, and land leachates	Carbonization, sol-gel polymerization, hydrothermal carbonization and acid mediated carbonization
Graphene oxide	Hydrophilic, electrical and thermal properties; high dispersity, compact structure	Filler in polymer nanocomposite materials, barrier to gas molecules (packing materials, protection of sensitive electronic devices and corrosion-resistant material). Sensors, photoluminescence, conductive inks, energy storage	Top down (extraction of graphene derivatives from graphite) and bottom-up (graphene is formed by carbon molecules) methods
Inorganics Not made of carbon or organic materials			
Metallics	Made of metal precursors, they can be mono-, bi-(core-shell), or poly-metallics; huge surface-to-volume ratio, ultraviolet–visible sensitivity, thermal, electrical, catalytic, and antibacterial properties; localized surface plasmon resonance; luminescence	Protection to plants and enhance food sustainability. Impacts on yield quality (germination rate, reproductive and vegetative characteristics). Biosensors	Physical, chemical, and biological techniques (chemical or biological reduction mainly)
Metallic-oxide	Made of positive metallic ions and negative oxygen ions; efficiency and reactivity enhanced	Nanofertilizers, nanopesticides; preserving, protecting, and extending the shelf stability of food; food packaging systems; nanosensors.	
Semiconductors	Nanocrystals (quantum dots); exhibit both metallic and non-metal-like properties. CdSe, CdTe, PbS	Water splitting, photonics, photocatalysis, and electronics; displays, solar cells, and bioimaging	
Ceramics	Inorganic solids made up of carbides, carbonates, or oxides	Agriculture (TiO_2_), catalysis, photo catalysis, photo degradation of dyes, and imaging applications	
Magnetics	Self-assemble properties (composites); biocompatibility, magnetic effects; classes: ferrites (metal oxides, v.g. Fe_3_O_4_, Fe_2_CoO_4_), ferrites with shell (v.g. drug, polymer, Au, metal oxide), metallic (Fe, Co, Ni), and metallic with a shell	Magneto-electrochemical immunosensors (label-based detection), electrochemical bioanalytical analysis, anti-biofouling (electrodes, F_3_O_4_), miRNA detection, hemoglobin (Hb) detection	
Organic NMs Composed of organic compounds (lipids or polymeric)			
Dendrimers	Polymer-based monodisperse macromolecules (1–20 nm); highly branched, symmetric branching units built around a core (active site); require further improvements in cytotoxicity profiles, biocompatibility, and biodistribution (due to number of generations or surfaces of the repeating units)	Vehicles for vaccines, genes, or drugs	Mainly two methods: 1—emulsification system and precipitation, gelification or polymerization; 2—precipitation of organic compounds in solution
Nanogels	Hydrophilic polymers (absorb water)	Carriers of pheromones and essential oils	
Nanocapsules	Core (substance) surrounded by a polymeric shell); toxicity reduced; two classes: liposomes (lipid bilayer separating an aqueous internal compartment from the bulk aqueous phase) and micelles (closed lipid monolayers with a fatty acid core and polar surface, or polar core with fatty acids on the surface)	Controlled release; liposomes: diagnosis and therapy, targeted drug delivery, and antimicrobial therapy; micelles: controlled release of substances (agrochemicals that are insoluble in water)	
Nanospheres	The substance is integrated into the polymer matrices	Protecting reservoirs and sustained release carriers (a longer duration of protection and a decrease in leaching losses (plants)	Others: spray-drying, supercritical fluid; piezoelectrical ways
Composites	NPs combined with at least one more type or phase of nanoparticles, or with bulk-type materials	Packaging materials and photothermal therapy (improve mechanical, thermal, and flame-retardant properties), drug delivery systems, antimicrobial wound dressings	Sol-gel, co-precipitation, hydrothermal synthesis, chemical bath deposition and in situ polymerization

**Table 2:** Nanomaterials used for the diagnosis of phytopathogenic fungi.

Nanomaterials	Bacteria	Recommended Dose	Reference
Platinum nanoparticles	*Bacillus thuringiensis* and *Bacillus subtilis*	*B. subtilis* from soil and root samples is detected to 10^7^ CFU mL^−1^	[64]
Gold nanoclusters (AuNCs, metallic nanomaterials that exhibit photoluminescence) added with three proteins, such as bovine serum albumin (BSA), human serum albumin (HSA), and lysozyme (Lys), and two metals, Zn^2+^ and Cadmium (Cd^2+^), produced AuNCs, Zn^2+^-Lys-AuNCs, Cd^2+^-Lys-AuNCs, Zn^2+^-BSA-AuNCs, Cd^2+^-BSA-AuNCs, Zn^2+^-HSA-AuNCs, and Cd^2+^-HSA-AuNCs	*E. coli*, and *Acetobacter aceti* *Pseudomonas aeruginosa*, *Bacillus*, *Bacillus natto*	The suspensions of the bacteria (OD_600_ = 0.05) generate a unique fluorescence response pattern when exposed to the array’s sensing elements	[65]
Gold nanoparticles loaded with copper and lead metal-organic frameworks with covalently immobilized specific DNA fragments onto the surface	*Salmonella typhimurium* and *Listeria monocytogenes*	The biosensor in real sample detection of *S. typhimurium* and *L. monocytogenes* with low LOD of 2.33 and 6.61 CFU mL^−1^, respectively	[66]

**Table 3:** Nanomaterials used for the diagnosis of phytopathogenic fungi.

Nanomaterial	Phytopathogenic Fungi	Description	Reference
CuNPs	*Sclerotinia sclerotiorum*	Gold electrodes were modified with Cu nanoparticles to detect salicylic acid	[72]
AuNPs	*Tilletia indica*	Immunosensor based on nanogold	[73]
	*Leptosphaeria maculans*	Combination of isothermal recombinase polymerase amplification (RPA) and gold nanoparticle-enhanced dynamic microcantilever (MCL) biosensor	[74]
	*Alternaria panax*	A colloidal gold particle-streptavidin (AuNPs-SA) conjugated lateral flow biosensor was used.	[75]
CdSe/ZnS	*F. oxysporum*	Use of 3-Mercaptopropionic acid-functionalized CdSe/ZnS QD in a fluorescence-based assay	[76]
Carbon nanostructures (CNSs)	*Aspergillus* sp. *Rhizopus* sp.	Identification of the possible appearance of fungi in strawberry fruits using carbon nanostructures	[77]

**Table 4:** Studies on the diagnosis of viral diseases in plants with the use of nanoparticles (NPs).

Nanosensor	Plant Material	Key Findings	Reference
Cadmium-telluride quantum dots biofunctionalized with the glutathione-S-transferase protein’s (GST) corresponding antibody (anti-GST)	Roots extracts (sugar beet)	High sensitivity and specificity (100%) of the immunosensor for the detection of *Polymyxa betae* (Keskin), the only vector of the beet necrotic yellow vein virus, which causes rhizomania in sugar beet.	[82]
AuNPs conjugated with thiolated probes	Leaf tissue	Detection of infection by the begomovirus genus in chili, tomato, bean, green chickpea, and black chickpea plants; high specificity of the nanosensor, with greater detection power than PCR.	[83]
Resonant quartz disk coated with immobilized specific antibodies	Raw saps (fresh flowers)	The immunosensor rapidly, specifically, and sensitively detects up to 1 ng of cymbidium mosaic potexvirus and odontoglossum ringspot tobamovirus using raw saps from infected orchids.	[84]
Antibody-conjugated cadmium teleride quantum dots (QD-Ab)	Plant sap (citrus trees leaves)	The immunobinding of QD-Ab with virus coat protein immobilized on the surface of carbon nanoparticles quenches the fluorescence of the quantum dots, allowing the detection of up to 220 ng × mL^−1^ of citrus tristeza virus.	[85]
Gold electrodes modified with antibody and AuNPs	Leaf extracts (*Nicotiana benthamiana*)	Detection of up to 10 pm/mL of plum pox virus in extracts of plum and tobacco leaves.	[86]

**Table 5:** Nanomaterials for the treatment of plants’ diseases caused by phytopathogenic bacteria.

Nanomaterials	Bacteria	Recommended Dose	Reference
For Bacteria Treatment			
Fullerene (C60), reduced graphene oxide (rGO), and multi-walled carbon nanotubes (MWCNTs)	Proteobacteria phylum	Applying 500 mg kg^−1^ of C60, rGO, and MWCNTs on the soil of a rice crop, the Proteobacteria phylum decreased by 2%, 9%, and 3.2%, respectively	[104]
Halloysite nanotube (a natural aluminosilicate nanotube) modified with cetyltrimethylammonium bromide (CTAB-Hal), sodium dodecyl sulfate (SDS-Hal), and Tween 80 (Tween 80-Hal)	*Agrobacterium tumefaciens*, *Xanthomonas oryzae*, and *R. solanacearum*	CTAB-Hal nanotube and Tween 80-Hal applied in vitro inhibited (killed) *A. tumefaciens* and *R. solanacearum* at 0.3 mg mL^−1^ and *X. oryzae* at 0.6 mg mL^−1^	[105]
Metal-based nanomaterials such as Cu, CuO, ZnO, calcium oxide (CaO), and magnesium oxide (MgO)	*Xanthomonas axonopodis* pv. punicae	The foliar application effectiveness was in the order Cu > ZnO > MgO > CuO > CaO with a minimum inhibitory concentration of 2.5, 20, 190, 200, and 1600 μg mL^−1^, respectively. Specifically, Cu-NPs killed *X. axonopodis* cells within 30 min at 2.5 μg mL^−1^	[106]
Cu-NPs added with 2 mmol Tween 20 (2.5 g) as surfactant	*Erwinia amylovora*, *X. campestris*, and *P. syringae*	*P. syringae* and X. campestris, 100 µg mL^−1^, and *E. amylovora*, 12 µg mL^−1^, in vitro and in vivo on plant pathogenic bacterial strains	[107]
Ag-NPs synthesized from *Solanum torvum* fruit extract	*X. axonopodis* pv. punicae and *R. solanacearum*	*X. axonopodis* pv. Punicae 6.25 μg mL^−1^ and *R. solanacearum* 12.5 μgmL^−1^, in vitro applications	[108]
Cu-NPs synthesized using *Azadirachta indica* as a reducing agent	*E. coli*	100 mg L^−1^ of Cu-NPs inhibited in 11 mm (zone of inhibition) the in vitro growth of *E. coli*	[109]

**Table 6:** Nanoparticles (NPs) used to control phytopathogenic fungi under in vivo conditions.

NPs	Description	Synthesis Method	Evaluation	Fungi	Effect	Reference
Ag	Silver nitrate (AgNO_3_) in tomato (*S. lycopersicum* L.)	Green synthesis	Greenhouse	*A. solani*	Resistance in plants by producing phenolics and other antioxidants.	[113]
Cu	Copper oxide nanoparticles (Cu_2_ONPs) in Cucumber (*C. sativus* L.)	Chemical synthesis	Greenhouse and field	*F. solani*	Irregularities, changes, twisting, and plasmolysis in the mycelia, as well as spore shrinking and collapsing in *F. solani.*	[114]
	Copper nanoparticles (Cu-NPs) on fruits of cucumber (*C. sativus*)	Green synthesis	Fruits of cucumber	*B. cinerea* and *S. sclerotiorum*	Reduction in incidence and severity.	[117]
Fe	Fe_2_O_3_ NP-Boron and Fe_2_O_3_ NP-Humic Acid in Cucumber	Synthesis in the presence of gamma-rays	In vivo	*F. oxysporum*	Reduction in disease indexes (20.8% and 25%). Promotion of systemic resistance.	[118]
Zn	ZnO-CuO NPs in *V. faba*	Myco-synthesis	Potted plants	*F. oxysporum*	Increase the yield parameters (pods/plant and pod weight, by 146.1% and 228.8%, respectively).	[119]
Ni	Ni_0.5_Al_0.5_Fe_2_O_4_ against dry rot of ginger	Hydrothermal method	Polyhouse in pots	*F. oxysporum*	Reduction in the incidence (28.2%).	[112]
S	Sulfur nanoparticles (S-NPs) on fruits of cucumber (*C. sativus*)	Precipitation reaction	Fruits of cucumber	*B. cinerea* and *S. sclerotiorum*	Low concentrations (5–100 µg/mL) reduced in incidence and severity.	[117]

**Table 7:** Selected studies on the treatment of viral diseases in plants with the use of nanoparticles (NPs).

Nanomaterial, Size, and Synthesis Method	Key Findings	Reference
AgNPs, 77–92 nm, biosynthesis (*Bacillus* spp. strains).	The AgNPs (synthesized from *Bacillus liqueniformis* and applied 24 h post-infection with bean yellow mosaic virus) prevented the destructive symptoms caused by the virus in broad bean leaves (yellow mosaic, mottling, wrinkling, size reduction, and deformation).	[123]
Commercial AgNPs (Sigma^®^).	The severity of the disease (tomato mosaic virus and potato Y virus) and the number of viruses decreased in tomato plants with the use of AgNPs; the NPs bound to the protein coat of the viral particles. Increase in photosynthetic pigments, total soluble protein, and peroxidase and polyphenol oxidase activity in treated plants.	[124]
AgNPs, 12.6 nm, biosynthesis (bacterial strains).	The application of AgNPs 24 h post-infection with tomato spotted wilt virus decreased the number of local lesions on *C. amarinticolor* and potato leaves. Less inhibitory effect when the application of AgNPs is carried out pre-infection with the virus. AgNPs induce systemic resistance and tissue changes (more spongy tissue, leaf thickness and length of vascular bundle).	[125]
AgNPs, 12 nm, chemical synthesis.	The application of AgNPs and salicylic acid 24 h post-infection with potato virus Y reduced the concentration of the virus and the percentage of infection in potatoes, an increase in the number and size of tubers, plant height, and amount of starch and soluble sugars.	[126]
AgNPs-graphene oxide, 30–50 nm, chemical synthesis.	The AgNPs applied post-infection decreased the virus concentration, the infection percentage, the symptoms, and the severity of the disease in lettuces inoculated with tomato bushy stunt virus.	[127]
ZnNPs, 100 nm, commercial product.	The symptoms and amount of cucumber mosaic virus were suppressed in eggplants treated with ZnNPs, 2-nitromethylphenol and *Ascophyllum nodosum* extract, under greenhouse conditions; absence of protein coat in viruses from treated plants, greater growth, yield, and amount of total and free phenols in treated plants.	[128]
ZnNPs, SiO_2_NPs, 18–20 nm, chemical synthesis.	Daily foliar spraying (pre-infection) of NPs on tobacco leaves for 12 d inhibited the replication of tobacco mosaic virus due to the activation of plant defense (accumulation of ROS, antioxidant enzymes, gene expression, and phytohormones).	[121]
Fe_3_O_4_NPs, biosynthesis (plants).	NPs are transported and accumulate in *N. benthamiana*, preventing the spread and proliferation of tobacco mosaic virus. Production of ROS, increased antioxidant activity, gene expression, and synthesis of phytohormones.	[129]
CeO_2_NPs.	NPs applied in vitro to *Datura stramonium* and *N. tabacum* leaves infected with tobacco mosaic virus inhibited the development of necrotic lesions and viral replication.	[130]
TiO_2_, 300–500 nm, chemical synthesis.	Broad bean plants treated with NPs (24 h pre-infection with broad bean stain virus) had a reduction in disease severity and greater growth; the regulatory and defense gene involved in the salicylic acid signaling pathway was expressed.	[120]

**Table 8:** Impact of NMs on environment and human health.

Nanomaterial/Synthesis Method	Key Findings	Reference
Human health		
TiO_2_ NPs/purchased NPs (Nanostructured and Amorphous Materials^®^)	TiO_2_ NPs generate reactive oxygen species, increase apoptosis and autophagy, and cause cytoplasmic translocation of the transcription factor Nrf2 to the nucleus in human neuroblastoma cell cultures.	[152]
SiO_2_, TiO_2_, Ag, polyacrylic acid (PAA)-coated cobalt ferrite/purchased NPs, co-precipitation	NPs caused toxicity (human neuroblastoma cell line SH-SY5Y in vitro) through membrane damage, cell cycle interference, reactive oxygen species (ROS) formation, and autophagosome accumulation.	[153]
Pt/Bioreduction (licopeno)	Decreased cell viability and proliferation in human acute monocytic leukemia (THP-1) macrophages; cytotoxicity (increased level of lactate dehydrogenase and intracellular protease), oxidative stress (ROS, MDA, nitric oxide, and carbonylated protein), activation of antioxidant system (glutathione, CAT, SOD, thioredoxin); cell death (mitochondrial dysfunction and decreased ATP levels, mitochondrial copies, and PGC-1α expression); altered gene expression, pro-inflammatory response.	[155]
ZnO and CuO NPs/not indicated	The released ions (Cu^2+^ and Zn^2+^) from textiles coated with ZnO and CuO NPs penetrate the epidermis (3D in vitro reconstructed epidermis model, EpiDerm^TM^) and dermal cells.	[156]
Environment		
Multi-Walled Carbon Nanotubes/purchased Sigma-Aldrich^®^	Alteration of cell morphology, membrane integrity, and mitochondrion function in *Allium cepa*. DNA damage, formation of micronuclei and chromosomal aberration, formation of internucleosomal fragments (apoptotic cell death), DNA methylation.	[157]
Poly (lactic-co-glycolic acid) NPs/chemical synthesis	In pure culture of nitrifying bacteria, ammonia oxidation was inhibited without significant effects on nitrite oxidation. In soil, the oxidation of both ammonia and nitrite was inhibited and the activity recovered after 14 days.	[158]
ZnO/chemical synthesis, nanoprecipitation	Greater neurotoxicity (zebrafish larvae and human neuroblastoma cells) with small sizes of NPs, symptoms similar to Parkinson’s disease (disruption of locomotor activity and gene expression), induction of dopaminergic neurons, loss and apoptosis in zebrafish brain, ROS production, mitochondrial damage and apoptosis, especially with long nanorod forms.	[159]
ZnO NPs/Ball milling and sol-gel	Spherical ZnO NPs (38 nm) at high concentrations (400 mg Zn/kg) caused oxidative stress (synthesis of H_2_O_2_, MDA, SOD, CAT, POX) in soybean compared to flower forms (59 nm) and rods (>500 nm).	[160]
Ag NPs/chemical reduction	Fluorescence quenching, accumulation of sugar and the reduction of Hill reaction activity in *Wolffia globose*; oxidative damage (increase in MDA content and SOD activity), decrease in the content of photosynthetic pigments and soluble protein.	[161]

MDA (malondialdehyde), SOD (superoxide dismutase), CAT (catalase), POX (guaiacol peroxidase).

## Data Availability

Data are contained within the article.
